# Familial Lymphoproliferative Malignancies and Tandem Duplication of NF1 Gene

**DOI:** 10.1155/2014/685857

**Published:** 2014-12-14

**Authors:** Gustavo Fernandes, Mirela Souto, Frederico Costa, Edite Oliveira, Bernardo Garicochea

**Affiliations:** Sirio Libanês Hospital, D. Adma Jafet, 91 Bela Vista, 01308-000 São Paulo, SP, Brazil

## Abstract

*Background*. Neurofibromatosis type 1 is a genetic disorder caused by loss-of-function mutations in a tumor suppressor gene (NF1) which codifies the protein neurofibromin. The frequent genetic alterations that modify neurofibromin function are deletions and insertions. Duplications are rare and phenotype in patients bearing duplication of NF1 gene is thought to be restricted to developmental abnormalities, with no reference to cancer susceptibility in these patients. We evaluated a patient who presented with few clinical signs of neurofibromatosis type 1 and a conspicuous personal and familiar history of different types of cancer, especially lymphoproliferative malignancies. The coding region of the NF-1 gene was analyzed by real-time polymerase chain reaction and direct sequencing. Multiplex ligation-dependent probe amplification was performed to detect the number of mutant copies. The NF1 gene analysis showed the following alterations: mosaic duplication of NF1, TRAF4, and MYO1D. Fluorescence in situ hybridization using probes (RP5-1002G3 and RP5-92689) flanking NF1 gene in 17q11.2 and CEP17 for 17q11.11.1 was performed. There were three signals (RP5-1002G3conRP5-92689) in the interphases analyzed and two signals (RP5-1002G3conRP5-92689) in 93% of cells. These findings show a tandem duplication of 17q11.2. *Conclusion*. The case suggests the possibility that NF1 gene duplication may be associated with a phenotype characterized by lymphoproliferative disorders.

## 1. Background

Neurofibromatosis type 1 is one of the most frequent autosomal dominant genetic disorders (OMIM 613113), affecting 1 in 3000 to 5000 people. It is caused by loss-of-function mutations in a tumor suppressor gene (NF1) mapped at 17q11.2 which codifies the protein neurofibromin [1]. Neurofibromin downregulates protooncogenes, like p21-ras, and therefore is involved in mechanisms of cell cycle control [2]. Various NF1 pseudogenes have been identified in the human genome. Duplication of internal part of these genes has been reported. Pseudogenes located in 2q21, 14q11, and 22q11 present a high sequence homology. It seems that a 640 kb fragment was originally duplicated in 2q21 and then transposed to 14q11. Subsequently a new duplication of this fragment in 14q11 was transposed to 22q11 [3]. Duplication of repetitive sequences is a not a feature restricted to pseudogenes, but it was also observed in NF1 gene by high resolution FISH [4]. Neurofibromatosis is phenotypically very heterogeneous and is associated with an increased susceptibility to different types of malignancies [5, 6].

The most conspicuous clinical manifestations of the syndrome are café-au-lait macules, axillary and inguinal freckling, iris hamartomas, peripheral neurofibromas, and osseous lesions. Soft tissue sarcomas, central nervous system neoplasms, neurofibrosarcomas, neuroendocrine tumors, and leukemias are among the most common associated malignancies [7].

The genetic alterations that modify neurofibromin function vary from discrete indels to the loss of the entire chromosomal segments containing the gene. A recent analysis of Slovak patients with neurofibromatosis type 1 revealed that the deletions and insertions were the most common abnormalities, being detected in almost 45% of cases. Duplications, in the other way, were rare, showing up in just 6% of patients. The phenotype in patients bearing duplication of NF1 gene was described so far to be restricted to bone and neurologic abnormalities. References to cancer in these individuals are rare [8].

We describe a case in which tandem duplications in NF1 gene were detected in a neurofibromatosis patient who presented a personal and familial history of compatible with a hereditary cancer syndrome, specially lymphoproliferative malignancies.

## 2. Case Presentation

We evaluated a 41-year-old male presented in September 2004 with an episode of acute gastrointestinal bleeding, which was associated with non-Hodgkin's lymphoma with a nonspecific subtype. The patient lived in a remote rural area in Northern Brazil and was promptly transferred to a larger center to receive chemotherapy to avoid a surgical procedure.

He was treated with three cycles of R-CHOP (rituximab, cyclophosphamide, doxorubicin, vincristine, and prednisone), without clinical response. He was then submitted to a surgical procedure and the anatomopathological study of retroperitoneal lymph nodes was consistent with an infiltrative carcinoma. The immunohistochemical analysis revealed chromogranin and synaptophysin expression and also a Ki 67 < 3%. At this moment carcinoid related markers (glucagon, gastrine, prolactin, PTH, 5HIAA, and chromogranin A) were normal. Because of the discordance between the two biopsies regarding the histological diagnoses, a review was performed in both specimens and there were no histological findings suggesting lymphoma and the hypothesis of a low grade neuroendocrine tumor was confirmed. The patient was then treated with octreotide acetate until clinical recovery and then, without any clinical or radiological evidence of disease, treatment was withheld.

On December 2010, he presented with an abdominal obstructive syndrome and an exploratory laparotomy was necessary. The anatomopathological diagnosis of mesenteric lymph nodes resected was consistent with areas containing marginal zone B-cell lymphoma/chronic lymphocytic leukemia and neuroendocrine tumor. Bone marrow was not infiltrated and FDG-PET showed exclusively abdominal nodal disease which was compatible with lymphoma involvement. At this time he was treated with 6 cycles of CHOP and octreotide was started again. CT scan obtained at the end of the treatment showed complete response.

The patient's family history was revealed to be very unusual. He referred to the fact that his father had a prostate cancer and the majority (if not all) of the 9 father brothers presented lymphoma or leukemia. These data could not be double checked since these individuals have died many years before in very distant places, but the fact that the patient was able to describe a very unique type of cancer in his relatives, many of them with low level of literacy, suggested that the presence of lymphoproliferative disorders is quite common in the paternal side of the family.

**Figure 1:**
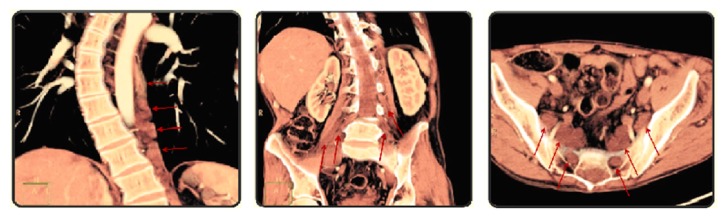
Abdominal CT scan showing neurofibromas.

At clinical examination the patient lacked any cutaneous stigma of neurofibromatosis, but one of the CT scans revealed the presence of various neurofibromas in the thorax and abdomen (Figure 1). He was tested for NF1 gene mutations and chromosomal abnormalities encompassing part of the long arm of chromosome 17 containing the NF1 gene.

The coding region of the NF-1 gene was analyzed by real-time polymerase chain reaction (RT-PCR) and direct sequencing. Multiplex ligation-dependent probe amplification (MLPA) was also performed to detect the number of mutant copies (deletions and duplications).

The following alterations were met: duplication of NF1, TRAF4, and MYO1D. Additionally, fluorescence in situ hybridization (FISH) using probes (RP5-1002G3 and RP5-92689) flanking the NF1 gene in 17q11.2 and CEP17 for 17q11.11.1 was performed. There were three signals (RP5-1002G3conRP5-92689) in the interphases analyzed and two signals (RP5-1002G3conRP5-92689) in 93% of cells. Those findings suggest that the patient had a tandem duplication of 17q11.2.

**Figure 2:**
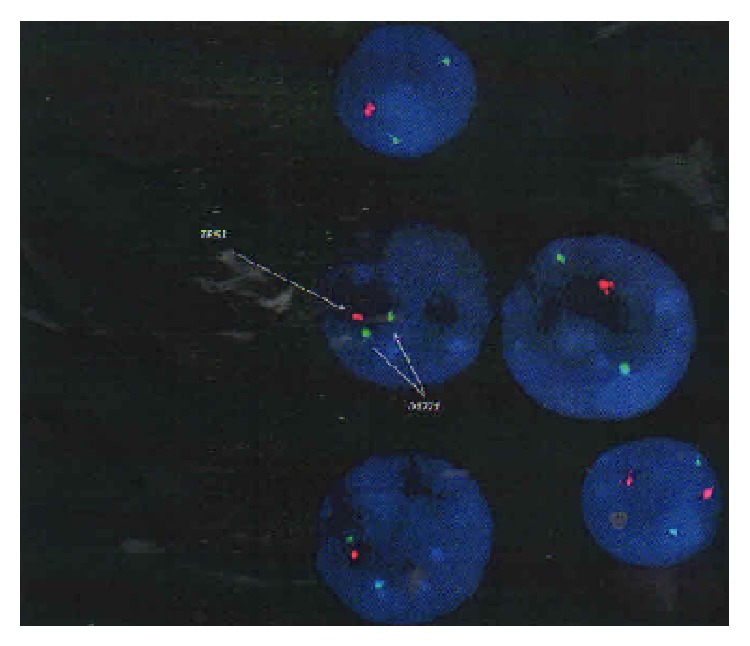
FISH analysis of TP53 (red signals).

On June 2013, the patient was submitted to routine laboratorial tests and a blood workout displayed a lymphocytosis (7500 lymphocytes/dL). The immunophenotype of the lymphocyte population was mostly composed of a clonal mature B lymphocytes positive for CD19, CD5, CD20, and CD23. These findings were consistent with CLL. FISH analysis displayed a del 17p13.1 (Figure 2).

The patient has been in treatment with octreotide acetate, without signals of disease progression. CLL is under clinical surveillance and the patient is out of treatment in the moment.

## 3. Discussion

It is well established that the incidence of tumors in patients with NF1 is high compared with the normal population, including malignant peripheral nerve sheath tumors (MPNST), optic gliomas and other gliomas, soft tissue sarcomas, gastrointestinal stromal tumors, breast cancer, and lymphomas/leukemias. The real incidence of cancer in this group is not well known [9]. Some studies have suggested that the relative risk of malignancy is around 4%, but it can range from 5% to 15%. This association is particularly relevant, in view of the increased morbidity and mortality, with a reduced life expectancy up to 15 years [6, 9].

There is some evidence that the NF1 gene product is a tumor suppressor, and when some mutation occurs, an uncontrolled cell proliferation is established. A reduction as a complete loss of neurofibromin induces RAS activation, as well as mitogen-activated protein kinase (MAPK), phosphatidylinositol 3-kinase (PI3K), protein kinase B (PKB), and mammalian target of rapamycin (mTOR) kinase. These pathways in turn can promote a cell growth beyond control [10, 11]. Other mechanism of tumor induction is due to loss of heterozygosity (LOH) or somatic mutations [12].

A retrospective study of a cohort of NF1 patients referred from UK-wide medical genetic centers showed that the most common germ line NF1 mutations were the following: frameshift mutations, splice-site mutations, nonsense mutations, missense mutations, and other less frequent types of mutation, including in-frame deletions, insertion/deletion mutations, single exon deletions, and intragenic multiexonic deletions [12].

The patient and their relatives have a strong history of cancer that is associated with other mutations, like deletions and insertions. However, there is no report correlating the presence of gene duplication with the presence of malignant neoplasms in NF1 patients, as we can see in this case.

A recent study had demonstrated that type-1 microdeletions, present in 5–10% of patients with neurofibromatosis type-1, increase the risk of MPNST in up 26% [13]. Regarding microduplications at 17q11.2, an analysis of seven patients with this feature showed that the most common clinical presentation is characterized by facial dysmorphisms, developmental delay, intellectual disability, and excessive neurofibromas. In spite of the broad spectrum of phenotypes, no history of malignancy was reported [14].

Recently, three reports have associated NF1 with developmental delay. White et al. [14] reported a three-year-old girl with a duplication in the NF1 gene, confirmed by FISH as a 17q11.2 duplication, which initially presented with delayed development, abnormal thyroid function, and elevated parathyroid hormone. There were no classic features of neurofibromatosis type 1. Caselli et al. [15] described a 6-year-old boy with psychomotor and language delay and other slight anomalies who displayed a 12.4 Mb duplication of 17q11.2q12. Finally, Bahuau et al. reported a child with developmental delay, low grade glioma, and aganglionic megacolon (Hirschsprung's disease). This unlikely combination of neurofibromatosis and intestinal neuronal dysplasia seemed to be the result of tandem duplication of NF1 gene and a reciprocal translocation, t(15;16) (q26.3;q12.1) [16].

Curiously, NF1 copy number variations are not among the most common genetic alterations associated with development delays. This was shown in an expanded map of almost 30.000 children suffering from developmental delay in which NF1 duplications were not described among the 70 most common copy number variations [17]. This data suggests that NF1 duplication is a very rare finding, that it can be associated with translocations and therefore associated with distinct syndromes, and that typical neurofibromatosis phenotype might not be so evident.

Finally it would be very interesting to look into what happens to both wildtype NF1 allele and the NF1 duplicated segment in the malignant tissue, such as lymphoma specimens and in the apparent neurofibromas. Unfortunately, the patient was asymptomatic when evaluated in our clinic, and no biopsy had been obtained from the neurofibroma which was diagnosed by MRI only. This investigation maybe would have enabled us to shed some light on what happens in the neurofibroma at both NF1 loci.

## 4. Conclusion

We presented a case in which a tandem duplicationof 17q11.2 was not associated with typical cutaneous findings of neurofibromatosis, but with neurofibromas. The patient displayed a conspicuous personal and familiar history of different types of cancer. Even though we could not have access to other members of the family to study the NF1 gene, the gene defection presented by the patient associated with many cases of lymphoproliferative disorders in the family suggests that the gene duplication can be part of a syndrome in which individuals are likely to develop hematologic malignancies. Since there is the lack of other similar published cases linking the presence of NF1 duplication with specific phenotypes, a comprehensive study about this type of mutation and its phenotype is warranted, mainly regarding the susceptibility to lymphoproliferative disorders. Opposed to other gene defects, like deletions and insertions, the real meaning of NF1 duplications and its role on cell cycle control remains unclear.

## Abbreviations

NF1: Neurofibromatosis type 1
CT: Computerized tomography
R-CHOPP: Rituximab + cyclophosphamide + hydroxydaunorubicin + vincristine + prednisone.
